# 

**Published:** 1883-06

**Authors:** 


					﻿Vol. IV.
JUNE, 1883.
No. 6.
THE
IndniniiIfiiiPraflihonre
yWoNTHLY Journal,
DEVOTED TO
MEDICINE, SURGERY, OBSTETRICS, DENTISTRY, PATHOLOGY
AND POPULAR SCIENCE.
W. c. BARRETT, M. D., D. D. S.,
EDITOR.
Columbia Publishing Compaq.
$2.50 Per Annum in Advance. -	- Single Copies, 25 Cents.
Entered at the Post-Office, Buffalo, N. Y., as Second-Class matter.
				

